# Contribution of Cancer-Targeting Drugs toward Faster Clinical Application

**DOI:** 10.3390/ijms23126445

**Published:** 2022-06-09

**Authors:** Sungpil Yoon, Hyung Sik Kim

**Affiliations:** School of Pharmacy, Sungkyunkwan University, 2066 Seobu-ro, Jangan-gu, Suwon 16419, Korea; hkims@skku.edu

With advances in cancer-targeting therapeutic strategies, cancer cells have developed drug resistance. For example, P-glycoprotein (P-gp) overexpression, various mutations in alternative pathways for growth signaling, or the evasion of apoptosis through mutations in related proteins such as the repair pathway could result in the recurrence of cancer cells, as well as resistance to cancer-targeting drugs. Moreover, cancers with poor prognosis are typically resistant to currently available cancer-targeting drugs, including advanced-stage cancers such as metastatic and stem cell-like cancers. Therefore, it is crucial to improve existing drugs and establish novel therapeutic protocols to overcome the failures of current cancer-targeting drugs. There are various therapeutic options and novel protocols for resistant cancers involving single drug and combination drug treatments, such as drug repositioning, compound derivatives, experimental drugs undergoing clinical trials, and plant extracts and natural products. To precisely target cancer cells, identifying underlying mechanisms or therapeutic protocols that could overcome the inefficiencies of current cancer-targeting drugs, along with rapid clinical applications, could afford superior treatment options for patients with drug-resistant cancers.

The faster clinical application of novel therapeutic protocols should be initiated by facilitating rapid clinical trials. To initiate faster clinical trials, investigators need to improve their knowledge of novel therapeutic protocols for cancer-targeting drugs in resistant cancer. Figure 1 summarizes how rapid clinical trials can be initiated to allow the application of novel therapeutic protocols for cancer-targeting drugs in resistant cancers. This can be divided into two parts. First, novel therapeutic protocols should be repeatedly assessed using various in vivo and in vitro assays, including xenograft models with cancer cell lines, orthotopic tumor models such as patient-derived xenograft mice or tumor engraftment, tumor spheroid assays, or repeated results with various other organ-originated cancer cell lines. In addition, the low toxicity of novel therapeutic protocols should be confirmed in normal cells using suitable animal models. Furthermore, protocols need to be evaluated in difficult-to-treat resistant cancer models, including P-gp-overexpressing, pancreatic, ovarian, and castration-resistant prostate cancers, as well as non-small cell lung cancer (NSCLC) and cancer stem cells. Accumulated and repeated data from various research groups could facilitate the verification of novel therapeutic protocols. These informative experimental results could facilitate the establishment of rapid clinical applications. Second, expediting clinical application could be accomplished by summarizing and evaluating numerous review studies and various existing informative literature studies on specific cancer-targeting drugs. By performing a meta-analysis of randomized controlled trials and observational studies, investigators can provide helpful suggestions for drugs considering cancer survival studies or prognosis data. A database analysis of cancer-targeting drugs could help to identify molecular targeting pathways or proteins in terms of changeable mRNA or protein database analysis. These endeavors would provide investigators with generalized concepts or ideas to treat specific drugs or tumors. Given the distinct genetic background of patients, database analysis can provide further detailed therapies for novel cancer-targeting drug protocols, personalized drug treatments, and finally, afford faster clinical applications of novel protocols for patients with resistant cancer (Figure 1).

Within this research topic, seven articles (five original research and two review articles) present novel protocols for faster clinical trials for cancer-targeting drugs and improved outcomes in patients with drug-resistant cancer. These articles also discuss better strategies to improve cancer-targeting drugs. These studies provide critical information needed for clinical trials, which could lead to therapeutic applications and the incorporation of these drugs in cancer treatment regimens. These findings will encourage the faster initiation of clinical trials, as well as therapeutic applications.

Four research articles discuss novel combination drug treatments to target various types of cancers which remain difficult to treat with cancer-targeting drugs. Oh et al. [1] report novel findings of combination treatment with a chemotherapeutic drug and JAK2 inhibitor for P-gp-overexpressing resistant cancer cells. The study focuses on a Food and Drug Administration (FDA)-approved JAK2 inhibitor by exploring a drug repositioning strategy. As the FDA provides easily accessible data on the beneficial and adverse effects of numerous drugs used in humans over a prolonged period, this could lower the costs and speed up the process of developing drugs to treat patients with drug-resistant cancer, as repeating a large number of toxicity tests could be circumvented [2,3]. Although JAK2 inhibitors have previously been reported to exhibit P-gp inhibitory activity, this study is the first to demonstrate that the FDA-approved JAK2 inhibitor fedratinib has P-gp inhibitory activity and a low dose can be combined with chemotherapeutic drugs. Based on this finding, the FDA-approved fedratinib, which can be repositioned to target cancer cells overexpressing P-gp, may lead to better treatment options in patients with drug-resistant cancer. Caylioglu et al. [4] examine another therapeutic combination strategy to treat glioblastoma (GBM), a difficult-to-treat chemo-resistant cancer. The authors demonstrate that co-treatment with AT101, a cottonseed-derived polyphenol gossypol, induced strong cytotoxicity against temozolomide (TMZ)-treated GBM cancer cells by inhibiting the p-p-42/44 signaling pathway. In particular, TMZ + AT101 co-treatment specifically targeted stem-like cells, which is a relapse model in brain cancer. TMZ + AT101 co-treatment increased cytotoxicity against stem-like cellular growth conditions known to be critical for glioblastoma recurrence. The authors speculated that TMZ + AT101 co-treatment could be a promising therapeutic strategy for GBM recurrence. The third combination therapy-related research report by Mrkvova et al. [5] investigates resistant T-cell leukemia Jurkat cells with mutations in apoptosis-related genes. The authors identify that co-treatment with tumor necrosis factor (TNF)-α and SMAC mimetic (LCL161) could induce RIP1-dependent necroptosis, re-activate apoptosis, and overcome apoptotic resistance. The authors conclude that this approach would be a suitable cancer-targeting strategy for overcoming treatment resistance in cells that fail to trigger apoptosis. The fourth combination therapy-related research article by Lee et al. [6] describes a novel technology for improving proton beam therapy in head and neck squamous cell carcinoma (HNSCC) models. The authors developed 3D tumor spheroid assay methods, which are reliable for evaluating combination therapy with protons and cancer-targeting drugs. Considering combination therapy with protons in HNSCC, olaparib, a PARP inhibitor, is known to target the DNA repair system. Using a 3D tumor spheroid assay system, the authors demonstrate that co-treatment with proton+olaparib might be an effective therapeutic protocol for HNSCC.

In the fifth research article, Kim et al. [7] present novel monotherapy findings with FDA-approved drug repositioning for human papillomavirus (HPV)-positive cervical cancer cells. The authors examine the anticancer potential of bazedoxifene, an FDA-approved drug for menopausal osteoporosis, in a specific HPV-positive cervical cancer tumor. Using both in vitro cell lines and in vivo xenograft models, bazedoxifene was found to inhibit cell growth via the GL130/STAT3 pathway and suppress the epithelial-to-mesenchymal transition capability of cancer cells. Given its FDA-approved status and documented toxicity in clinical settings, bazedoxifene could be more easily applied to clinical patients with HPV-positive cervical cancers. Considering that this study targeted HPV-positive cervical cancer cells, a specific type of tumor, it can be deemed as a personalized medicine and narrowed down to specific drug treatment. As fedratinib and bazedoxifene are currently applied in clinical settings [1,7], drug repositioning offers an efficient method to address the urgent need for the pharmacological treatment of drug-resistant cancer, affording treatment approval at a relatively rapid pace.

As mentioned earlier, review studies are also important for the rapid application of novel cancer-targeting drugs in clinical trials. Two review articles examine the present topic. Hyun and Shin [8] focus on KRAS as a cancer-targeting molecule exhibiting specificity, given that KRAS is mutated in poor prognosis-related cancers such as NSCLC and pancreatic cancers. The authors discuss a novel technology-based treatment that targets KRAS using targeted protein degradation (TPD) strategies with proteolysis-targeting chimeras. It is suggested that TPD-based small-molecule chemicals could overcome drug resistance and KRAS mutation-mediated evasion with current cancer-targeting drugs. The authors speculate that this strategy could be a promising therapeutic option in patients with KRAS mutations. Another review article by Halubiec [9] focuses on synthetic retinoids as castration-resistant prostate cancer-targeting drugs. Derivatives of natural retinoids have potential against this resistant form of prostate cancer. The authors summarize various experimental studies using synthesized retinoids within recent decades, including their structures and specifically targeted molecules. These review articles have sparked the interest of investigators to examine retinoids as potential castration-resistant prostate cancer-targeting drugs.

Identifying novel cancer-targeting protocols (with single-drug or combination therapies) to target resistant cancer cells could help to overcome the shortcomings of current cancer-targeting drugs and afford better treatment options for patients with drug-resistant cancers. The seven articles in this Special Issue highlight the different aspects of cancer-targeting protocols in resistant cancers and provide information on the development of improved treatment strategies. The novel cancer-targeting protocols discussed herein will facilitate the initiation of clinical trials and lead to faster therapeutic applications for patients with resistant cancers.

## Figures and Tables

**Figure 1:**
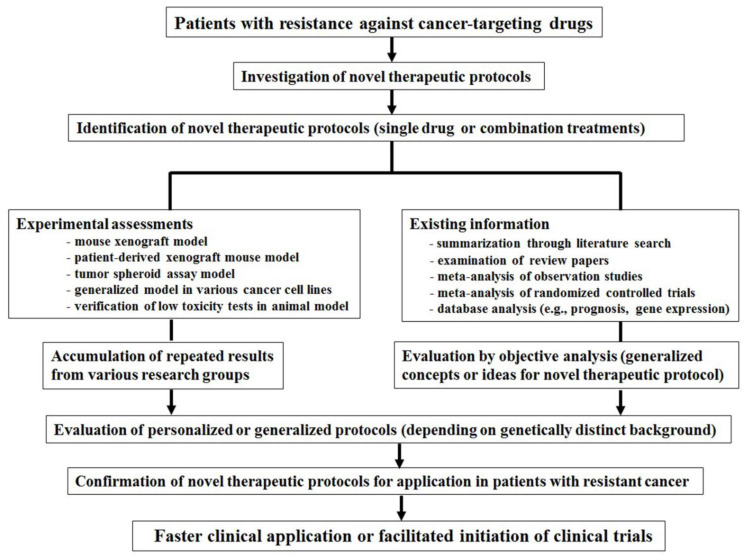
Scheme summarizing the initiation of faster clinical trials to apply novel therapeutic protocols for cancer-targeting drugs against resistant cancer. To initiate faster clinical trials, completing experimental tests and reviewing existing information can increase the possibility of initiating faster clinical applications for novel therapeutic protocols. Repeated and accumulated results will support the rapid clinical application of novel therapeutic protocols for cancer-targeting drugs against resistant cancer.
